# 1,1′-[(2-Bromo­phen­yl)­methyl­ene]­dipyrrolidin-2-one

**DOI:** 10.1107/S1600536812006277

**Published:** 2012-02-17

**Authors:** Hong-Qi Li, G. Ramachandran, V. Satheesh, K. Sathiyanarayanan, R. S. Rathore

**Affiliations:** a Key Laboratory of Science and Technology of Eco-Textiles, Ministry of Education, College of Chemistry, Chemical Engineering and Biotechnology, Donghua University, Shanghai 201620, People’s Republic of China; b Chemistry Division, School of Advanced Sciences, VIT University, Vellore 632 014, India; c Bioinformatics Infrastructure Facility, Department of Biotechnology, School of Life Sciences, University of Hyderabad, Hyderabad 500 046, India

## Abstract

In the title compound, C_15_H_17_BrN_2_O_2_, both pyrrolidinone rings adopt envelope conformations. The crystal packing is characterized by short C—Br⋯O=C inter­actions [Br⋯O = 3.1730 (13) Å], leading to supra­molecular dimers. Inter­molecular C—H⋯O and C—H⋯π inter­actions are also observed.

## Related literature

For a related structure, see: Camus *et al.* (2001 ▶). For related references on Br⋯O inter­actions, see: Allen *et al.* (1997 ▶); Damodharan *et al.* (2004 ▶).
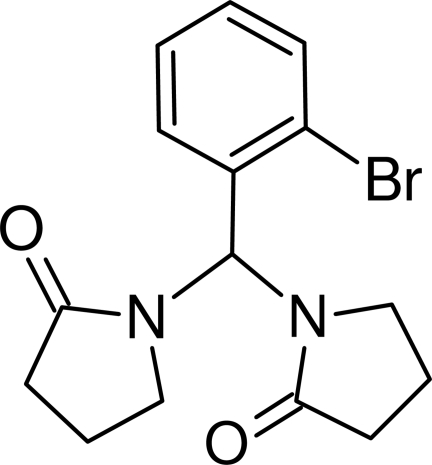


## Experimental

### 

#### Crystal data

C_15_H_17_BrN_2_O_2_
*M* *_r_* = 337.22
Monoclinic, 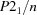

*a* = 7.9734 (3) Å
*b* = 11.0788 (4) Å
*c* = 15.9456 (6) Å
β = 91.859 (1)°
*V* = 1407.82 (9) Å^3^
*Z* = 4
Mo *K*α radiation
μ = 2.92 mm^−1^
*T* = 123 K
0.20 × 0.18 × 0.18 mm

#### Data collection

Bruker APEXII CCD area-detector diffractometer
Absorption correction: multi-scan (*SADABS*; Bruker, 2004 ▶) *T*_min_ = 0.593, *T*_max_ = 0.621
15532 measured reflections
4296 independent reflections
3661 reflections with *I* > 2σ(*I*)
*R*_int_ = 0.027

#### Refinement

*R*[*F* ^2^ > 2σ(*F* ^2^)] = 0.025
*wR*(*F* ^2^) = 0.057
*S* = 1.01
4296 reflections
181 parameters
H-atom parameters constrained
Δρ_max_ = 0.43 e Å^−3^
Δρ_min_ = −0.38 e Å^−3^

### 

Data collection: *APEX2* (Bruker, 2004 ▶); cell refinement: *SAINT-Plus* (Bruker, 2004 ▶); data reduction: *SAINT-Plus*; program(s) used to solve structure: *SHELXS97* (Sheldrick, 2008 ▶); program(s) used to refine structure: *SHELXL97* (Sheldrick, 2008 ▶); molecular graphics: *ORTEP-3* (Farrugia, 1997 ▶); software used to prepare material for publication: *SHELXL97* and *PLATON* (Spek, 2009 ▶).

## Supplementary Material

Crystal structure: contains datablock(s) global, I. DOI: 10.1107/S1600536812006277/xu5471sup1.cif

Structure factors: contains datablock(s) I. DOI: 10.1107/S1600536812006277/xu5471Isup2.hkl

Supplementary material file. DOI: 10.1107/S1600536812006277/xu5471Isup3.cml

Additional supplementary materials:  crystallographic information; 3D view; checkCIF report

## Figures and Tables

**Table 1:** Hydrogen-bond geometry (Å, °) *Cg* is the centroid of the C10–C15 ring.

*D*—H⋯*A*	*D*—H	H⋯*A*	*D*⋯*A*	*D*—H⋯*A*
C2—H2*B*⋯O2^i^	0.97	2.40	3.363 (2)	174
C6—H6*B*⋯O2^i^	0.97	2.59	3.528 (2)	163
C11—H11⋯O1^i^	0.93	2.56	3.328 (2)	140
C13—H13⋯O2^ii^	0.93	2.55	3.223 (2)	130
C8—H8*B*⋯*Cg*^iii^	0.97	2.75	3.6405 (19)	152

Symmetry codes: (i) 
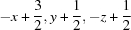
; (ii) 
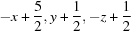
; (iii) 
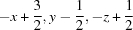
.

**Fig. 1.**
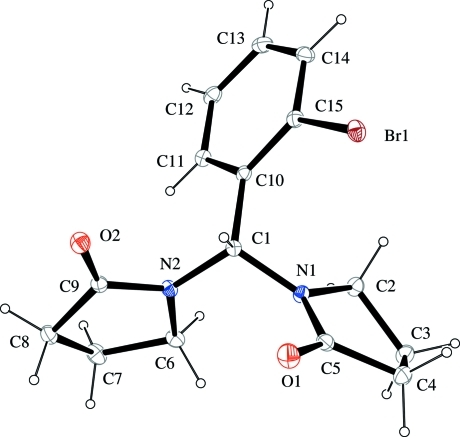
A view of (I) with non-H atoms shown as probability ellipsoids at 30% levels (Farrugia, 1997). H atoms radii are on an arbitrary scale.

**Fig. 2.**
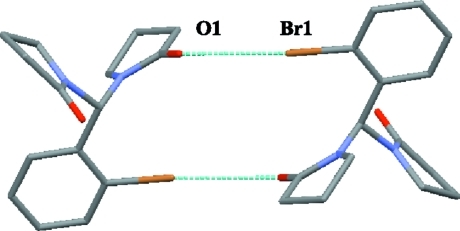
Molecular dimers via Br···O interaction.

## supplementary crystallographic information

### Comment

In the title compound, C_15_H_17_BrN_2_O_2_, both pyrrolidinone rings (N1/C2—C5; N2/C6—C9) adopt envelope conformation with C3 and C7 atoms at their flap, respectively (Fig. 1). Crystal packing is characterized by C15—Br1···O1—C5^i^ interaction [symmetry code (i): 2 - *x*,1 - *y*,-*z*; Br···O = 3.1730 (13) Å, C—Br···O = 170.33 (5)°] leading to molecular dimers (Fig. 2). Intermolecular C—H···O and C—H···π interactions additionally stabilize the packing (Table 1).

### Experimental

In a dry 100 ml Erlenmeyer flask 2-pyrrolidone (10 mmol), benzaldehyde (10 mmol), iodine (15 mol %) and dichloromethane (DCM; 15 ml) were taken. The reaction mixture was stirred at room temperature (25°C) for one hour. The reaction was monitored by TLC and after the completion of reaction iodine utilized was set free from the product by treating it with aqueous sodium thiosulfate solution and extracted into DCM (2 *X* 20 ml). The crude reaction mixture was purified by column chromatography on silica gel using ethyl acetate/hexane as the eluents. Final yields: 82%; m.p. 354 (1)°K. Suitable single crystals for data collection were grown from ethanol and tetrahydrofuran (THF) mixture (1:1).

### Refinement

H atoms were placed in the geometrically expected positions and refined with the riding options. The calculated distances with hydrogen atoms are: C(*sp*^2^)—H = 0.93 Å, *C*(methylene)—H = 0.97 Å, C(methine)—H = 0.98 Å and *U*_iso_ = 1.2 *U*_eq_(parent).

### Crystal data

**Table:**

C_15_H_17_BrN_2_O_2_	*F*(000) = 688
*M**_r_* = 337.22	*D*_x_ = 1.591 Mg m^−^^3^
Monoclinic, *P*2_1_/*n*	Melting point: 354(1) K
Hall symbol: -P 2yn	Mo *K*α radiation, λ = 0.71073 Å
*a* = 7.9734 (3) Å	Cell parameters from 840 reflections
*b* = 11.0788 (4) Å	θ = 2.1–24.0°
*c* = 15.9456 (6) Å	µ = 2.92 mm^−^^1^
β = 91.859 (1)°	*T* = 123 K
*V* = 1407.82 (9) Å^3^	Block, colorless
*Z* = 4	0.20 × 0.18 × 0.18 mm

### Data collection

**Table:**

Bruker APEXII CCD area-detector diffractometer	4296 independent reflections
Radiation source: fine-focus sealed tube	3661 reflections with *I* > 2σ(*I*)
Graphite monochromator	*R*_int_ = 0.027
φ and ω scans	θ_max_ = 30.6°, θ_min_ = 2.2°
Absorption correction: multi-scan (*SADABS*; Bruker, 2004)	*h* = −11→11
*T*_min_ = 0.593, *T*_max_ = 0.621	*k* = −14→15
15532 measured reflections	*l* = −22→22

### Refinement

**Table:**

Refinement on *F*^2^	Primary atom site location: structure-invariant direct methods
Least-squares matrix: full	Secondary atom site location: difference Fourier map
*R*[*F*^2^ > 2σ(*F*^2^)] = 0.025	Hydrogen site location: inferred from neighbouring sites
*wR*(*F*^2^) = 0.057	H-atom parameters constrained
*S* = 1.01	*w* = 1/[σ^2^(*F*_o_^2^) + (0.0228*P*)^2^ + 0.7592*P*] where *P* = (*F*_o_^2^ + 2*F*_c_^2^)/3
4296 reflections	(Δ/σ)_max_ = 0.002
181 parameters	Δρ_max_ = 0.43 e Å^−^^3^
0 restraints	Δρ_min_ = −0.38 e Å^−^^3^

### Special details

**Table:**

Geometry. All e.s.d.'s (except the e.s.d. in the dihedral angle between two l.s. planes) are estimated using the full covariance matrix. The cell e.s.d.'s are taken into account individually in the estimation of e.s.d.'s in distances, angles and torsion angles; correlations between e.s.d.'s in cell parameters are only used when they are defined by crystal symmetry. An approximate (isotropic) treatment of cell e.s.d.'s is used for estimating e.s.d.'s involving l.s. planes.
Refinement. Refinement of *F*^2^ against ALL reflections. The weighted *R*-factor *wR* and goodness of fit *S* are based on *F*^2^, conventional *R*-factors *R* are based on *F*, with *F* set to zero for negative *F*^2^. The threshold expression of *F*^2^ > σ(*F*^2^) is used only for calculating *R*-factors(gt) *etc*. and is not relevant to the choice of reflections for refinement. *R*-factors based on *F*^2^ are statistically about twice as large as those based on *F*, and *R*- factors based on ALL data will be even larger.

### Fractional atomic coordinates and isotropic or equivalent isotropic displacement parameters (Å^2^)

**Table:**

	*x*	*y*	*z*	*U*_iso_*/*U*_eq_	
C1	0.86332 (17)	0.68852 (12)	0.18659 (8)	0.0138 (3)	
H1	0.9215	0.6106	0.1871	0.017*	
C2	0.75434 (19)	0.81209 (13)	0.05820 (9)	0.0182 (3)	
H2A	0.8600	0.8379	0.0356	0.022*	
H2B	0.7103	0.8762	0.0925	0.022*	
C3	0.62857 (19)	0.77622 (15)	−0.01195 (9)	0.0218 (3)	
H3A	0.5146	0.7938	0.0037	0.026*	
H3B	0.6515	0.8186	−0.0636	0.026*	
C4	0.6548 (2)	0.64047 (14)	−0.02201 (9)	0.0223 (3)	
H4A	0.5493	0.6001	−0.0355	0.027*	
H4B	0.7329	0.6238	−0.0659	0.027*	
C5	0.72546 (18)	0.60005 (14)	0.06225 (9)	0.0178 (3)	
C6	0.58936 (18)	0.75537 (13)	0.25328 (9)	0.0180 (3)	
H6A	0.5089	0.7226	0.2124	0.022*	
H6B	0.6087	0.8398	0.2406	0.022*	
C7	0.5283 (2)	0.73979 (15)	0.34296 (10)	0.0231 (3)	
H7A	0.5544	0.8108	0.3765	0.028*	
H7B	0.4080	0.7264	0.3425	0.028*	
C8	0.62238 (18)	0.62932 (14)	0.37781 (9)	0.0191 (3)	
H8A	0.6604	0.6428	0.4355	0.023*	
H8B	0.5511	0.5584	0.3756	0.023*	
C9	0.76951 (17)	0.61425 (12)	0.32157 (8)	0.0151 (3)	
C10	0.99547 (17)	0.78631 (13)	0.19871 (8)	0.0139 (2)	
C11	0.98318 (18)	0.87298 (13)	0.26151 (9)	0.0169 (3)	
H11	0.8926	0.8708	0.2968	0.020*	
C12	1.10341 (19)	0.96235 (14)	0.27229 (9)	0.0201 (3)	
H12	1.0935	1.0188	0.3150	0.024*	
C13	1.23820 (19)	0.96825 (14)	0.21989 (10)	0.0220 (3)	
H13	1.3175	1.0293	0.2267	0.026*	
C14	1.25446 (18)	0.88301 (14)	0.15741 (10)	0.0200 (3)	
H14	1.3442	0.8867	0.1217	0.024*	
C15	1.13587 (18)	0.79196 (13)	0.14840 (9)	0.0161 (3)	
N1	0.77437 (15)	0.69982 (10)	0.10618 (7)	0.0147 (2)	
N2	0.74562 (15)	0.68714 (10)	0.25438 (7)	0.0146 (2)	
O1	0.73991 (15)	0.49624 (10)	0.08795 (7)	0.0253 (2)	
O2	0.89131 (13)	0.54791 (10)	0.33318 (7)	0.0210 (2)	
Br1	1.171997 (18)	0.670483 (13)	0.066302 (9)	0.01925 (5)	

### Atomic displacement parameters (Å^2^)

**Table:**

	*U*^11^	*U*^22^	*U*^33^	*U*^12^	*U*^13^	*U*^23^
C1	0.0145 (6)	0.0132 (6)	0.0136 (6)	0.0009 (5)	0.0010 (5)	0.0000 (5)
C2	0.0219 (7)	0.0148 (7)	0.0178 (6)	0.0008 (5)	−0.0014 (5)	0.0013 (5)
C3	0.0200 (7)	0.0269 (8)	0.0184 (7)	0.0013 (6)	−0.0022 (5)	0.0011 (6)
C4	0.0262 (8)	0.0241 (8)	0.0166 (7)	−0.0066 (6)	0.0000 (6)	−0.0032 (6)
C5	0.0182 (6)	0.0188 (7)	0.0166 (6)	−0.0046 (5)	0.0040 (5)	−0.0032 (5)
C6	0.0148 (6)	0.0160 (7)	0.0233 (7)	0.0036 (5)	0.0030 (5)	0.0013 (5)
C7	0.0227 (7)	0.0219 (8)	0.0252 (8)	0.0032 (6)	0.0089 (6)	−0.0016 (6)
C8	0.0208 (7)	0.0197 (7)	0.0171 (6)	−0.0026 (6)	0.0044 (5)	−0.0004 (5)
C9	0.0175 (6)	0.0125 (6)	0.0154 (6)	−0.0029 (5)	0.0006 (5)	−0.0011 (5)
C10	0.0146 (6)	0.0133 (6)	0.0136 (6)	0.0007 (5)	−0.0014 (5)	0.0015 (5)
C11	0.0179 (7)	0.0174 (7)	0.0155 (6)	0.0002 (5)	0.0000 (5)	0.0000 (5)
C12	0.0238 (7)	0.0169 (7)	0.0191 (7)	−0.0015 (6)	−0.0049 (5)	−0.0020 (5)
C13	0.0181 (7)	0.0195 (7)	0.0279 (8)	−0.0034 (6)	−0.0049 (6)	0.0025 (6)
C14	0.0135 (6)	0.0227 (8)	0.0238 (7)	0.0003 (6)	0.0011 (5)	0.0049 (6)
C15	0.0157 (6)	0.0167 (6)	0.0160 (6)	0.0033 (5)	0.0000 (5)	0.0013 (5)
N1	0.0178 (6)	0.0123 (5)	0.0140 (5)	−0.0013 (4)	−0.0008 (4)	−0.0006 (4)
N2	0.0142 (5)	0.0140 (6)	0.0158 (5)	0.0018 (4)	0.0029 (4)	0.0015 (4)
O1	0.0359 (6)	0.0154 (5)	0.0246 (6)	−0.0066 (5)	0.0020 (5)	−0.0017 (4)
O2	0.0206 (5)	0.0194 (5)	0.0229 (5)	0.0047 (4)	0.0015 (4)	0.0052 (4)
Br1	0.02074 (8)	0.01928 (8)	0.01803 (7)	0.00452 (6)	0.00520 (5)	−0.00019 (6)

### Geometric parameters (Å, º)

**Table:**

C1—N1	1.4504 (17)	C7—C8	1.531 (2)
C1—N2	1.4545 (17)	C7—H7A	0.9700
C1—C10	1.5192 (19)	C7—H7B	0.9700
C1—H1	0.9800	C8—C9	1.5088 (19)
C2—N1	1.4664 (18)	C8—H8A	0.9700
C2—C3	1.530 (2)	C8—H8B	0.9700
C2—H2A	0.9700	C9—O2	1.2272 (17)
C2—H2B	0.9700	C9—N2	1.3501 (18)
C3—C4	1.528 (2)	C10—C11	1.393 (2)
C3—H3A	0.9700	C10—C15	1.3997 (19)
C3—H3B	0.9700	C11—C12	1.385 (2)
C4—C5	1.508 (2)	C11—H11	0.9300
C4—H4A	0.9700	C12—C13	1.384 (2)
C4—H4B	0.9700	C12—H12	0.9300
C5—O1	1.2251 (19)	C13—C14	1.382 (2)
C5—N1	1.3589 (18)	C13—H13	0.9300
C6—N2	1.4569 (18)	C14—C15	1.387 (2)
C6—C7	1.535 (2)	C14—H14	0.9300
C6—H6A	0.9700	C15—Br1	1.9059 (14)
C6—H6B	0.9700		
			
N1—C1—N2	110.43 (11)	C8—C7—H7B	110.7
N1—C1—C10	111.61 (11)	C6—C7—H7B	110.7
N2—C1—C10	111.99 (11)	H7A—C7—H7B	108.8
N1—C1—H1	107.5	C9—C8—C7	104.72 (12)
N2—C1—H1	107.5	C9—C8—H8A	110.8
C10—C1—H1	107.5	C7—C8—H8A	110.8
N1—C2—C3	102.63 (11)	C9—C8—H8B	110.8
N1—C2—H2A	111.2	C7—C8—H8B	110.8
C3—C2—H2A	111.2	H8A—C8—H8B	108.9
N1—C2—H2B	111.2	O2—C9—N2	124.65 (13)
C3—C2—H2B	111.2	O2—C9—C8	127.11 (13)
H2A—C2—H2B	109.2	N2—C9—C8	108.25 (12)
C4—C3—C2	104.12 (12)	C11—C10—C15	117.22 (13)
C4—C3—H3A	110.9	C11—C10—C1	121.22 (12)
C2—C3—H3A	110.9	C15—C10—C1	121.55 (12)
C4—C3—H3B	110.9	C12—C11—C10	121.16 (13)
C2—C3—H3B	110.9	C12—C11—H11	119.4
H3A—C3—H3B	109.0	C10—C11—H11	119.4
C5—C4—C3	104.31 (12)	C13—C12—C11	120.45 (14)
C5—C4—H4A	110.9	C13—C12—H12	119.8
C3—C4—H4A	110.9	C11—C12—H12	119.8
C5—C4—H4B	110.9	C14—C13—C12	119.72 (14)
C3—C4—H4B	110.9	C14—C13—H13	120.1
H4A—C4—H4B	108.9	C12—C13—H13	120.1
O1—C5—N1	124.68 (14)	C13—C14—C15	119.48 (14)
O1—C5—C4	127.23 (13)	C13—C14—H14	120.3
N1—C5—C4	108.08 (12)	C15—C14—H14	120.3
N2—C6—C7	103.15 (12)	C14—C15—C10	121.90 (13)
N2—C6—H6A	111.1	C14—C15—Br1	117.84 (11)
C7—C6—H6A	111.1	C10—C15—Br1	120.24 (11)
N2—C6—H6B	111.1	C5—N1—C1	120.62 (12)
C7—C6—H6B	111.1	C5—N1—C2	113.36 (12)
H6A—C6—H6B	109.1	C1—N1—C2	125.19 (11)
C8—C7—C6	105.16 (11)	C9—N2—C1	121.21 (11)
C8—C7—H7A	110.7	C9—N2—C6	114.74 (11)
C6—C7—H7A	110.7	C1—N2—C6	123.90 (11)
			
N1—C2—C3—C4	−26.53 (15)	C1—C10—C15—Br1	−3.48 (18)
C2—C3—C4—C5	25.03 (15)	O1—C5—N1—C1	5.5 (2)
C3—C4—C5—O1	166.88 (15)	C4—C5—N1—C1	−173.64 (12)
C3—C4—C5—N1	−14.00 (16)	O1—C5—N1—C2	175.60 (14)
N2—C6—C7—C8	−19.18 (15)	C4—C5—N1—C2	−3.55 (16)
C6—C7—C8—C9	18.77 (16)	N2—C1—N1—C5	−91.10 (15)
C7—C8—C9—O2	168.80 (14)	C10—C1—N1—C5	143.64 (12)
C7—C8—C9—N2	−11.29 (16)	N2—C1—N1—C2	100.04 (15)
N1—C1—C10—C11	115.42 (14)	C10—C1—N1—C2	−25.22 (18)
N2—C1—C10—C11	−8.97 (18)	C3—C2—N1—C5	19.40 (16)
N1—C1—C10—C15	−65.71 (16)	C3—C2—N1—C1	−171.04 (12)
N2—C1—C10—C15	169.89 (12)	O2—C9—N2—C1	2.8 (2)
C15—C10—C11—C12	1.3 (2)	C8—C9—N2—C1	−177.13 (12)
C1—C10—C11—C12	−179.76 (13)	O2—C9—N2—C6	178.53 (13)
C10—C11—C12—C13	0.8 (2)	C8—C9—N2—C6	−1.39 (16)
C11—C12—C13—C14	−1.2 (2)	N1—C1—N2—C9	141.19 (13)
C12—C13—C14—C15	−0.5 (2)	C10—C1—N2—C9	−93.76 (15)
C13—C14—C15—C10	2.7 (2)	N1—C1—N2—C6	−34.15 (17)
C13—C14—C15—Br1	−175.86 (11)	C10—C1—N2—C6	90.89 (15)
C11—C10—C15—C14	−3.0 (2)	C7—C6—N2—C9	13.31 (16)
C1—C10—C15—C14	178.04 (13)	C7—C6—N2—C1	−171.07 (12)
C11—C10—C15—Br1	175.43 (10)		

### Hydrogen-bond geometry (Å, º)

Cg is the centroid of the C10–C15 ring.

**Table:**

*D*—H···*A*	*D*—H	H···*A*	*D*···*A*	*D*—H···*A*
C2—H2*B*···O2^i^	0.97	2.40	3.363 (2)	174
C6—H6*B*···O2^i^	0.97	2.59	3.528 (2)	163
C11—H11···O1^i^	0.93	2.56	3.328 (2)	140
C13—H13···O2^ii^	0.93	2.55	3.223 (2)	130
C8—H8*B*···*Cg*^iii^	0.97	2.75	3.6405 (19)	152

Symmetry codes: (i) −*x*+3/2, *y*+1/2, −*z*+1/2; (ii) −*x*+5/2, *y*+1/2, −*z*+1/2; (iii) −*x*+3/2, *y*−1/2, −*z*+1/2.
